# Glycemic control among children and adolescents with diabetes in Southern Ethiopia: a cross-sectional study

**DOI:** 10.1186/s12902-022-01070-y

**Published:** 2022-06-15

**Authors:** Mulugeta Sitot Shibeshi, Alemneh Kabeta Daba, Kebede Mola Meiso, Birkneh Tilahun Tadesse

**Affiliations:** 1 Department of Pediatrics and Child Health, Hawassa University, Hawassa, Ethiopia; 2 School of Nursing, Hawassa University, Hawassa, Ethiopia; 3 Department of Pediatrics and Child Health, Alatyon General Hospital, Hawassa, Ethiopia

**Keywords:** Adolescents, Children, Diabetes, Ethiopia, Glycemic control

## Abstract

**Background:**

Glycemic control is an important part of diabetes management. Strict glycemic control has been shown to reduce the long-term complications of diabetes. However, achieving good glycemic control is challenging for people with diabetes especially in resource limited settings. The aim of this study was to assess glycemic control and identify its determinants among children and adolescents with diabetes.

**Methods:**

A cross-sectional study among 116 children and adolescents with diabetes was done at a pediatric endocrine clinic in southern Ethiopia. Data on socioeconomic, demographic, nutrition, and diabetes related variables were collected. Glycemic control was assessed based on glycosylated hemoglobin level. Logistic regression analysis was used to identify predictors of glycemic control.

**Results:**

The mean glycated hemoglobin (HbA1c) of the participants was 9.6 ± 2.4% (81 ± 3 mmol/mol). Ninety seven (83.6%) of the study participants had poor glycemic control [HbA1c ≥7.5% (58 mmol/mol)]. The presence of lipodystrophic change at injection sites (*p* =0.028) and being from a family that cannot afford for insulin when there is no free supply (*p* =0.009) were associated with poor glycemic control.

**Conclusions:**

The majority of children and adolescents with diabetes had poor glycemic control. Stakeholders shall focus on identifying strategies to improve the magnitude of poor glycemic control. More research is warranted to exhaustively list out factors contributing to poor glycemic control.

## Background

Diabetes is the most common endocrine-metabolic disorder of childhood and adolescence with important consequences on physical and emotional development [1]. The incidence of type 1 diabetes has been on the rise worldwide [2, 3]. The morbidity and mortality associated with diabetes are due to its acute metabolic derangements and long-term complications such as retinopathy, nephropathy, neuropathy, ischemic heart disease, and arterial obstruction with gangrene of the extremities [1].

Although several factors predispose children to chronic complications of diabetes, the strongest modifiable predictor is glycemic control [4, 5]. The Diabetes Control and Complication Trial (DCCT) showed that strict glycemic control delayed the onset and slowed the progression of chronic complications of diabetes [6]. A reliable index of long-term glycemic control is provided by measurement of glycated hemoglobin (HbA1c) as it measures the glycemic control over the preceding 2 – 3 months [1]. Consequently, measurement of HbA1c is often recommended to be done every 3 months. However, the frequency of measurement should depend on the clinical situation, the treatment plan and the judgment of the clinician [7]. According to the recommendations of the American Diabetic Association and the International Society for Pediatric and Adolescent Diabetes (ISPAD), children and adolescents who have access to comprehensive diabetes care should aim to achieve a target of HbA1c < 7.0% (53 mmol/mol). However, a higher HbA1c goal (in most cases < 7.5% [58 mmol/mol]) is appropriate in settings where there is lack of access to analog insulins, advanced insulin delivery technology, ability to regularly check blood glucose, and continuous glucose monitoring [8, 9].

Studies from Africa have consistently shown poor glycemic control in children and adolescents with type 1 diabetes. A recent study in Sudan showed that 76% of children and adolescents with diabetes had poor glycemic control [10]. Similarly, studies in Cameroon [11] and Tanzania [12] showed higher average HbA1c values than the recommended target. Compared to reports from Africa, lower mean HbA1c levels were observed in eight high income countries in Europe and North America [13].

In Ethiopia, children and adolescents with diabetes receive donated insulin and blood glucose test strips. However, parents must purchase them out-of-pocket when there are shortages, and this could cost them about 600 Ethiopian Birr per month (about12.69 USD as per the exchange rate on November 14, 2021).

Children and adolescents < 15 years with diabetes are cared for by pediatricians or general practitioners (if there are no pediatricians); and those diagnosed with diabetes before the age of 15 years would continue to be cared for by pediatricians until the age of 18 years. On the other hand, adolescents aged > 14 years at diagnosis of diabetes are cared for by internists or general practitioners in the absence of internists.

A study conducted in Ethiopia documented that more than half (52.3%) of children and adolescents with diabetes had poor glycemic control. However, the HbA1c cutoff used to define poor glycemic control in that study was HbA1c > 10% (86 mmol/mol) which was higher than the recommended target; and the study did not identify factors associated with glycemic control [14]. Therefore, this study aimed to assess glycemic control and identify its predictors among children and adolescents with diabetes on regular follow-up at a pediatric endocrine clinic in southern Ethiopia.

## Methods and materials

### Description of the study area

The study was conducted at the endocrine clinic of Hawassa University Comprehensive Specialized Hospital located about 275 km south of Addis Ababa, the capital city of Ethiopia. The pediatric endocrine clinic operates 2 days per week to evaluate children and adolescents with diabetes on regular follow-up. To date, 230 diabetic children and adolescents have follow-up in the clinic. During each visit, patients are evaluated for glycemic control, acute and chronic complications, growth and development, and are provided with diabetic education and insulin.

### Study design, subjects and sample

The study was a hospital based cross-sectional study among children and adolescents with diabetes age ≤ 18 years. Children and adolescents with diabetes who have been on insulin treatment for ≥3 months and who had HbA1c tests were included in the study. Those who had no HbA1c tests or who have been on insulin treatment for < 3 months were excluded. Hence, out of the 230 children and adolescents with diabetes on follow-up, only 116 were enrolled in the study.

### Data collection

Patients’ medical records were reviewed, patients and caregivers were interviewed, and information pertinent to their diabetes was recorded on a pretested data collection tool by trained nurses when patients came to the endocrine clinic for their routine follow-up visits.

The data collection tool contained questions about sociodemographic and diabetes related information. Sociodemographic information including age, sex, address, family structure, primary caregiver, age of primary caregiver, level of parental/caregivers’ education, parental/ caregivers’ occupation, income of the family, ability of the family to buy insulin when there is no free supply, and family history of diabetes were collected. Data on diabetes related information including age at diagnosis, condition at initial presentation, duration of illness, insulin regimen, frequency of insulin injection, total daily dose of insulin [sum of all Neutral Protamine Hagedorn Insulin (NPH) and Regular Insulin (RI) given over 24 hours (unit/kg/day)], adherence to treatment, caregiver involvement in diabetes related tasks, means of insulin storage, lipodystrophic changes at injection sites, anthropometric measurements, number of meals per-day, and presence of diabetic complications (acute and chronic) were also collected. Acute complications included history of severe hypoglycemia at home and diabetic ketoacidosis (DKA). The presence of chronic complications of diabetes (retinopathy, nephropathy and neuropathy) was checked by reviewing medical records of the study subjects. In the diabetic clinic, children were screened for chronic complications based on the screening criteria of the American Diabetes Association [8]. The presence of diabetic retinopathy was evaluated using fundal photography. Diabetic neuropathy was assessed by screening for symptoms/signs of neuropathy and doing comprehensive foot exam. However, screening for nephropathy was made only by urine dipstick test for proteinuria as urinary albumin–to–creatinine ratio or any other early marker of albuminuria is not available in this setting*.*

Anthropometric parameters were measured using standard procedures. The presence of lipodystrophy was assessed by examining insulin injection sites. The results of the most recent point-of-care HbA1c tests (measured using in2it™ (I) Analyzer, Bio-Rad Laboratories Deeside, CH5 2NU, UK) were taken from the patients’ medical records. As resources are limited and comprehensive care is not currently provided in our setting, we used a less- stringent HbA1c goal (< 7.5% [58 mmol/mol]) to classify patients as having good or poor glycemic control. Hence, an HbA1c value of < 7.5% (58 mmol/mol) was taken as indicator of good glycemic control while a value of ≥7.5% (58 mmol/mol) was considered to be indicator of poor control [8, 9].

### Data management and analysis

Data were entered in to Statistical Package for Social Sciences (SPSS) software (version 20) for windows and cleaned. Descriptive and analytic statistics were computed. Nutritional status was determined using weight for height, body mass index for age, and height for age Z- scores that were generated using WHO Anthro and WHO Anthro plus softwares. Sociodemographic characteristics and diabetes related variables were summarized using descriptive statistics. Mean or median and standard deviation (SD) or interquartile range (IQR) were calculated for continuous data. Bivariate logistic regression analysis was carried out to determine candidate variables (with a *p*-value < 0.25) for the multivariable logistic regression model. On the multivariable logistic regression analysis *p*-values of less than 0.05 were considered statistically significant. Results from the bivariate and multivariable analyses are presented using a table.

## Results

Among a total of 230 children and adolescents with diabetes, 111 had no point-of-care HbA1c tests as the analyzer was not functional (and they could not afford to have the tests done in a private health institution) and 3 had duration of illness of < 3 months. Hence, only 116 children and adolescents with diabetes, aged 21 months to 18 years, participated in the study. The mean age of the participants was 13.1 ± 4.6 years. The median age at diabetes diagnosis was 10.5 years (Interquartile range [IQR]: 7.3 – 12.0), and the median duration since diabetes diagnosis was 3 years (IQR: 1.0 – 5.0). The sociodemographic characteristics of children and adolescents with type 1 diabetes are presented in Table 1.

**Table 1:** Sociodemographic characteristics of children and adolescents with type 1 diabetes (*N* = 116)

Characteristics	Results
	N(%)
Age ≥ 10 yrs	92 (79.3)
Female	60 (51.7)
Nutritional status	
Normal	83 (71.6)
Under weight	22 (19)
Over weight	10 (8.5)
Obese	1 (0.9)
Residence (urban)	70 (60.3)
Primary care giver’s education	
No formal education	32 (27.6)
Primary/secondary school	70 (60.3)
Higher education	14 (12.1)
Parents living together	90 (77.6)
Monthly family income (≥2000ETB)^a^	61 (52.6%)
Family able to afford to buy insulin (No)	81 (69.8)
Number of main meals per day(> 3)	67 (57.8)
Family history of diabetes (Yes)	23 (19.8)

^a^Median (IQR) = 2000ETB (700 – 5000 ETB); ETB = Ethiopian Birr; 1USD = 47.29ETB (on November 14, 2021)

**Fig. 1:**
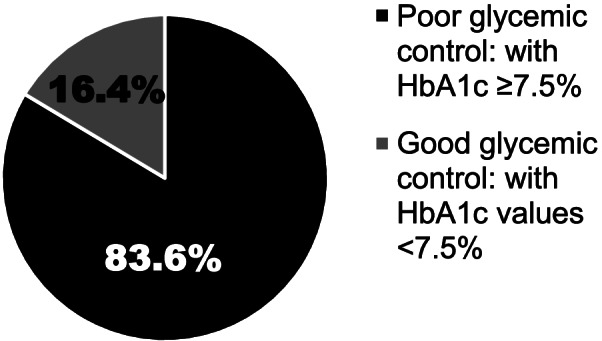
Proportion of children and adolescents with type 1 diabetes with good or poor glycemic control (*N* = 116)

The mean HbA1c was 9.6 ± 2.4% (81 ± 3 mmol/mol) [pre-adolescent (age < 10 yrs): 9.2 ± 2.3% (77 ± 2 mmol/mol) and adolescent (age ≥ 10 yrs): 9.8 ± 2.5% (84 ± 4 mmol/mol)]. The overall prevalence of poor glycemic control was 83.6% (Fig. 1). Eight (0.07%) and nine (0.08%) children had emergency admissions for severe hypoglycemia and DKA, respectively during their follow-up. Fifty-three (45.7%) study subjects had refrigerators to store their insulin (although longer power failures are frequent in our region) while 60 patients (51.7%) used any household item, usually a plastic cup with cover, that can hold small amount of sand and cold water to store their insulin, and 3 patients kept their insulin at room temperature. The median total daily dose of insulin (unit/kg/day) was 0.9(IQR: 0.7-1.1) although the dose was individualized as it was titrated against the patient’s glycemic control during the course of follow-up. Most children were on twice daily injections (98.3%) and a few were on one injection per day. No child was using multiple daily injections although 16.4% of children used additional insulin for high blood sugar levels. None of the study subjects used premixed insulin. Most of the study subjects (83.6%) reported that they did not miss any insulin dose during the 3 months period prior to the data collection. All of the study subjects used syringes and needles (no insulin pens) to administer insulin, and all of them reported multiple times re-use of disposable insulin syringes without sterilization. However, no infection at injection sites was reported. Insulin regimen and injection frequency are presented in Table 2. Sixty-nine children (75.8%) had primary caregivers with formal education of different levels. Among the cases that fulfilled the American Diabetes Association criteria of screening for chronic complications of diabetes [8], one patient had diabetic retinopathy (out of 37 children screened), no one had symptoms and signs of neuropathy, and none had proteinuria on urine dipstick test.

**Table 2:** Diabetes related characteristics of children and adolescents with type 1 diabetes (*N* = 116)

Characteristics	Category	Frequency	
		N	**%**
Initial presentation	DKA	94	81
	Symptoms of DM	21	18.1
	Incidental	1	0.9
Duration of diabetes, in years	< 5	88	75.8
	≥5	28	24.2
Insulin regimen	NPH	12	10.3
	NPH + RI	104	89.7
Insulin injection frequency	Once	2	1.7
	Twice	114	98.3
Additional insulin for high blood sugar levels	Yes	19	16.4
	No	97	83.6
Frequency of self-monitoring of blood glucose in a day	Once	5	4.3
	2 times	92	79.3
	3 times	17	14.7
	4 times	2	1.7
Parental supervision in those who measure blood glucose and administer insulin themselves (*n* = 85)	Always	31	36.5
	Occasional	46	54.1
	Never	8	9.4
Lipodystrophic changes at injection sites	Yes	33	28.4
	No	83	71.6

*DKA* Diabetic Ketoacidosis, *NPH* Neutral Protamine Hagedorn Insulin, *RI* Regular Insulin, *DM* Diabetes Mellitus

On the basis of the results from the bivariate logistic regression analysis, lipodystrophic change at insulin injection sites, being from a family that cannot afford to buy insulin when there is no free supply, having 3 or fewer meals in a day, and educational status of the primary caregiver were candidates for the multivariable analysis. On multivariable logistic regression analysis lipodystrophic change at injection sites and not being able to afford for insulin when there is no free supply were associated with poor glycemic control. Children and adolescents with lipodystrophic change at injection sites were about four times [3.98 (95% CI: 1.16, 13.21), p. =0.028] more likely to have poor glycemic control; and those from families that cannot afford for insulin when there is no free supply were about six times [5.98 (95%CI: 1.58, 22.73), p. =0.009] more likely to have poor glycemic control (Table 3).

**Table 3:** Logistic regression analysis on factors associated with poor glycemic control among children and adolescents with type 1 diabetes (*N* = 116)

Characteristics	Category	COR (95%CI), ***p***.	AOR (95%CI), ***p***.
Can parents afford for insulin when there is no free supply?	Yes	1	1
	No	5.00 (1.65, 15.15), 0.004	5.98 (1.58, 22.73), 0.009
Lipodystrophic change at insulin injection site	Yes	3.11 (1.03, 9.37), 0.044	3.98 (1.63, 13.21), 0.028
	No	1	1
Average number of meals in a day	≤3	3.17 (1.98, 10.26), 0.054	2.13 (0.55, 8.16), 0.27
	≥4	1	1
Primary caretaker ever attended formal education	No	3.0 (1.64, 14.02), 0.16	1.64 (0.25, 10.77), 0.61
	Yes	1	1

*COR* Crude Odds Ratio, *AOR* Adjusted Odds Ratio, *CI* Confidence Interval; p.: *p*-value

## Discussion

This study assessed the magnitude of poor glycemic control and identified associated factors among children and adolescents with diabetes on regular follow-up at a pediatric endocrine clinic in southern Ethiopia. The result showed poor glycemic control in the majority of the study subjects (83.6%). Being from a family that cannot afford for insulin when there is no free supply and presence of lipodystrophic changes at insulin injection sites were associated with poor glycemic control. The mean HbA1c [9.6 ± 2.4% (81 ± 3 mmol/mol)] in this study is higher than the recommended target [< 7.5%% (58 mmol /mol)]; and it is comparable to findings in other resource limited settings [10–12]. However, the mean HbA1c observed in this study is higher than findings from high income countries [13, 15] although our adolescent mean HbA1c of 9.8 ± 2.5% (84 ± 4 mmol/mol) is not so far from the United States data in 15-18 year olds of 9.3% (78 mmol/mol)( [16]). This variation in glycemic control could be due to differences in socioeconomic status as poor socioeconomic status contributes to poor glycemic control through its undesired effects on quality of diabetes care, treatment adherence, and provision of adequate and healthy nutrition [17].

Some of the predictors of glycemic control in children in the literature include diabetes duration and insulin dose [15], age [12], care givers’ involvement in blood glucose monitoring [11], lipodystrophic changes at injection sites [18, 19], and diet quality [20]. In this study, being from a family that cannot buy insulin when there is no free supply and presence of lipodystrophy at insulin injection sites were identified as predictors of poor glycemic control. Children and adolescents with lipodystrophy were four times more likely to have poor glycemic control than those without it; and those who came from families that cannot afford for insulin were six times more likely to have poor glycemic control than their counterparts.

Although all study subjects reported that they had regularly rotated their injection sites, 33 children (28.4%) had lipodystrophy, suggesting that other factors such as reuse of needles might have played a role in its development [21]. The prevalence of lipodystrophy at injection sites observed in this study (28.4%) is lower than that reported in a previous study conducted in Ethiopia (56.8%) [18]. Our finding is also lower than findings from Egypt (54.9%) [22] and Spain (64.4%) [23]. The statistically significant association between the presence of lipodystrophy at injection sites and poor glycemic control observed in our study was also reported by several studies [18, 19, 24, 25]. Injection of insulin into a lipodystrophic site may lead to its erratic absorption which could contribute for poor glycemic control [26]. The improvement in glycemic control seen with rotation of injection sites indicates that lipodystrophic changes impair insulin absorption from such sites [27].

The median duration of diabetes in our study is short (3 years). This is partly because of the increase in the number of referred patients to our diabetic clinic as our hospital is a referral hospital in the region with a relatively better care provision. The rate of DKA at initial diagnosis in this study (81%) is much higher than those reported from the United States (34%) [28], New Zealand (47.4%) [29], and Brazil (58.8%) [30], but lower than another study from Ethiopia (96.5%) [14]. The reason for the very high incidence of DKA at initial presentation in our setting could be, although it needs to be studied, low awareness about diabetes. Although we do not know if some have died of DKA undiagnosed, there have not been records of deaths from DKA at diagnosis or during follow-up. The finding in this study is not consistent with reports from other developing countries where mortality from DKA was high (Kenya,9.6%) [31] and (India, 13.2%) [32].

The DCCT demonstrated that the use of three or more daily injections improved glycemic control. However, no child was using multiple daily injections (MDI) of insulin in our setting. This calls for the need to move to MDI as part of the interventions to improve glycemic control [33].

The majority (69.8%) of families could not afford to buy insulin when there is no free supply. One possible solution to address this problem would be promoting the enrollment of families to the Community-Based Health Insurance (CBHI) scheme. CBHI is a health insurance that pools members’ premium payments (385 Ethiopian Birr [about $8.1] and 510 Ethiopian Birr [$10.8]/ household/year for rural and urban dwellers, respectively) into a collective fund, which is managed by the members and covers basic health care costs at governmental health institutions when a member is sick. This scheme will help families save money (that would otherwise be used to cover health care expenses of family members) to buy insulin when there is no free supply.

The management of diabetes should be based on guidelines such as those of the American Diabetes Association “Standards of Medical Care in Diabetes” and ISPAD. However, the cost of this management is expensive for developing countries and the care provided is sub-optimal in these settings [34, 35]. While working towards establishing guidelines-based care, low-income countries should try to provide affordable care that result in better clinical outcomes. Because of the significant variations in the quality of care provided to children and adolescents with diabetes among countries at different resource levels, Graham D. Ogle et al. developed a Levels of Care concept with three tiers [35]. According to this Levels of Care concept, the care provided in our setting would mostly lie in the Minimal Care tier (level 1C) although some components of Intermediate Care are also available [self-monitoring of blood glucose, diabetes education, and screening for some of the complications (retinopathy and neuropathy)]. Hence, concerted effort to fully implement the Intermediate Care is needed to improve treatment outcome.

The cross-sectional nature of the study design and being conducted at a single follow-up site with the participation of limited number of children and adolescents with diabetes can be considered as limitation of the study. As some of the data used in the study were self-reported by patients and/or their caregivers, recall and desirability biases that could affect some of the results might have occurred. Participation of a larger number of children and adolescents with diabetes from multiple follow-up centers would have allowed identification of more predictors of glycemic control.

## Conclusions

Poor glycemic control was prevalent in children and adolescents with diabetes. Being from a family that cannot afford for insulin when there is no free supply and presence of lipodystrophic changes at insulin injection sites contributed to poor glycemic control. Regular monitoring of glycemic control shall be strengthened. On top of this, strategizing mechanisms to sustain insulin supply and taking measures to reduce development of lipodystrophy shall also be considered. This study determined the magnitude of poor glycemic control, identified some of the associated factors, and revealed some of the gaps in the care of children and adolescents with diabetes. We hope that the findings of this study would be an input to improve practice and provide optimal care for children with diabetes in our region and in other similar settings. We recommend more multicenter studies with a larger number of participants.

## Data Availability

The datasets used and/or analysed during the current study are available from the corresponding author on reasonable request.

## Abbreviations

HbA1c: Glycated hemoglobin A1c
DCCT: Diabetes Control and Complication Trial
ISPAD: International Society for Pediatric and Adolescent Diabetes
MDI: Multiple daily injections
SPSS: Statistical Package for Social Sciences
WHO: World Health Organization
IQR: Inter quartile range
ETB: Ethiopian Birr
DKA: Diabetic ketoacidosis
DM: Diabetes mellitus
NPH: Neutral Protamine Hagedorn Insulin
RI: Regular Insulin
AOR: Adjusted odds ratio
CI: Confidence interval
